# Proarrhythmic risk and determinants of cardiac autonomic dysfunction in collagen-induced arthritis rats

**DOI:** 10.1186/s12891-016-1347-6

**Published:** 2016-11-29

**Authors:** Ting-Tse Lin, Yen-Ling Sung, Chih-En Wu, Hong Zhang, Yen-Bin Liu, Shien-Fong Lin

**Affiliations:** 1 Department of Internal Medicine, National Taiwan University Hospital Hsin-Chu Branch, Hsinchu, Taiwan; 2 Institute of Biomedical Engineering, National Chiao-Tung University, 1001 Ta-Hsueh Road, Hsinchu, 300 Taiwan; 3 Department of Electrical Engineering, Xi’an Jiaotong University, Xi’an, Shaanxi China

**Keywords:** Autonomic function, Collagen-induced arthritis, Rheumatoid arthritis, Heart rate variability, Deceleration capacity

## Abstract

**Backgrounds:**

Patients with rheumatoid arthritis (RA) have increased risk of sudden cardiac death (SCD), which is two-fold higher than general population. The driving cause of SCD was considered due to lift-threatening arrhythmia where systemic inflammation acts as the pathophysiological basis linking RA to autonomicdysfunction.

**Methods:**

To assess the sympathetic over-activity of “inflammatory reflex”, we measured heart rate variability (HRV) in a rat collagen-induced arthritis (CIA) model, whose arthritis is induced in Lewis rats by intradermal injection of emulsion of type II collagen. Single-lead electrocardiogram (ECG) was recorded for 30 min every two days. Time and frequency-domain parameters, detrended fluctuation analysis (DFA), deceleration (DC) and acceleration capacity (AC) were analyzed.

**Results:**

Compared with 9 control rats, many of HRV parameters of 9 CIA rats revealed significant different. At the beginning of arthritis, LF/HF was significant higher than controls (1st week: 2.41 ± 0.7 vs. 1.76 ± 0.6, *p* < 0.05; 2nd week: 2.24 ± 0.5 vs. 1.58 ± 0.5, *p* < 0.05) indicating intensive inflammatory reflex at the initial phase of inflammation but no significant difference was observed in the following recover phase. The similar trend of DFA parameters was noted. However, the DC appeared progressive lower despite of no significant increase of the LF/HF compared with controls since 4th week.

**Conclusions:**

We observed sympathetic over-activation of inflammatory reflex during early stage of arthritis in CIA rats. The ongoing decline of DC indicated advanced cardiac autonomic dysfunction regardless of remission of acute arthritis.

**Electronic supplementary material:**

The online version of this article (doi:10.1186/s12891-016-1347-6) contains supplementary material, which is available to authorized users.

## Background

Rheumatoid arthritis (RA) is a common autoimmune disease that is associated with progressive disability, systemic complications, early cardiac death and socioeconomic costs. RA is characterized by loss of tolerance to self-proteins that contain a citrulline residue, where anti-citrulline response can be initiated with T-cell, B-cell and tumor necrosis factor α (TNF-α). Synovitis is launched and perpetuated by positive feedback loops and in turn promotes systemic disorders, including myocardial infarction, stroke and heart failure [1]. The cardiac mortality is 1.5-fold higher and sudden cardiac death (SCD) is twice as likely among RA patients than general population. Although the main focus of clinical setting is on accelerated atherosclerosis and myocardial injury, chronic systemic inflammation may increase arrhythmogenicity via other mechanisms and contribute to higher risk of SCD [2]. Synovitis derived inflammatory cytokines (interleukin-1, interleukin-6 and TNF-α) could induce non-structural myocardium change via direct electrophysiological effect on cardiomyocyte and sympathetic overactivation [3]. Many studies demonstrated inflammatory cytokines could prolong action potential duration via changing expression and function of potassium and calcium channel [4–6]. On the other hand, signs of autonomic dysfunction in RA population have been documented decades ago and involved cardiovascular nervous system [7]. Heart rate variability (HRV) is a non-invasive, practical and reproducible method to assess the effect of sympathovagal balance on the heart. In general population, reduced HRV indicates increased sympathetic tone and decreased parasympathetic system being associated with higher risk of cardiovascular events [8]. Several studies investigating HRV in RA patients, reported a depression of time- and/or frequency-domain measures of HRV, implying an increase in the sympathetic drive of the heart rate in this population. We intend to test the hypothesis that “inflammatory reflex” could be a mechanism leading to heart impairment in RA [9].

## Methods

Collagen-induced arthritis (CIA) in rat is an animal model for rheumatoid arthritis (RA) and can be induced in Lewis rat. The CIA model can be used to unravel mechanisms involved in the development of arthritis and frequently used to study the effect of new therapeutics.

### Animals

Eighteen adult Lewis rats weighing 160–180 g were used. The animals were housed under special pathogen-free conditions at the animal facility. These rats were divided into 2 groups: CIA and control group equally. The healthy condition and physical activity of the rats were monitored daily, and the body weight and paw thickness were measured and recorded every week.

### Induction of arthritis

Chick Type II collagen (CII) (Chondrex cat. #20012) was dissolved in 0.05 M acetic acid by gently stirring overnight at 4 °C. Equal amount collagen and complete Freud’s Adjuvant (CFA) (Chondrex cat. #7024) were mixed in an ice-water bath, adding the collagen drop-wise to the CFA while mixing. The same procedure was conducted when mixing Bovine Type II collagen (Chondrex cat. #20022) with Incomplete Freud;s Adjuvant (IFA) (Chondrex cat.#7002) On day 0, 0.5 ml of the emulsion (containing 0.5 mg Chick CII + CFA) was injected at the base of the tail of each rat. On day 7, a second injection (0.5 mg Bovine CII + IFA) was administered in the same way. For arthritis assessment, all rats were monitored three times a week by the same person blinded to the treatment group, and the clinical scores were evaluated. Zero score represents no edema or swelling in the joint; 1 score represents slight edema and erythema limited to the foot and/or ankle; 2 scores represent edema and erythema toes and most joints of the ankles; 3 scores represent severe edema and erythema paw below ankle joint; 4 scores represent edema and erythema of all paws including ankle joint [10]. The cumulative score for all four paws of each rat (maximum possible score of 16) was used to represent the overall disease severity and progression. The model of arthritis was considered successful when the scores were greater than 5.

### NN Interval recordings

The ECG of CIA rats was recorded for 30 min every two days (CIA rats and controls). Prior to ECG recordings, animals were conditioned for 7 consecutive days, 30 min in each morning (0700–0900 h) on a self-made platform (Additional file 1: Figure S1). The self-made platform was designed to prevent rats to strip off recording electrodes in non-anesthetized condition. A day before ECG was firstly recorded, the ventral thoracic region of each animal was carefully shaved. We placed the negative electrode near the right shoulder and the positive electrode to the left of the xyphoid space to obtain robust P, R, S, and T waves, as human lead II configuration. We kept the rat awaken and recorded ECG signal using a bioamplifier. The ECG signal was analyzed using custom-made LabVIEW software which automatically detected the R-waves. The QRS complexes were automatically and manually classified as normal sinus rhythm, atrial and ventricular premature beats. The term “NN interval”, or the so-called normal-to-normal intervals isthe intervals between adjacent QRS complexes resulting from sinus node depolarizations. ECGs were bandpass filtered (2–300 Hz) and, after R wave peak detection, 180-s tachograms were generated. The RR intervals were deduced from adjacent normal sinus beats and transferred to a personal computer to be post-processed by a Matlab program (Kubios HRV) [11].

### Time and frequency domain parameters

The mean heart rate, standard deviation of N-N intervals (SDNN) and root mean square of successive differences of N-N interval (RMSSD) were used as time-domain measures of HRV. The power spectrum densities were estimated by Welch’s averaged periodogram method [12].

The LF power (0.04 to 0.15 Hz) and HF power (0.15 to 0.4 Hz) were derived from the sum of area within specific frequency range under the power spectrum density curve of the entire 30-min segment. The normalized unit (NU) of HF (HF NU) or LF (LF NU) was calculated as the ratio of the absolute powers of HF or LF to TP and multiplied by 100.

Generally, SDNN reflects overall heart rate fluctuation. RMSSD could represent parasympathetic tone and also be sensitive to uneven beat detection. In terms of frequency domain, LF is considered to reflect the combined modulation of efferent vagal and efferent sympathetic nervous system activity and HF is related to modulation of efferent vagal activity by ventilation. LF/HF ratio is often claimed to characterize “sympathovagal balance” or “relative sympathetic activity” because the LF reflects modulation by both the sympathetic and parasympathetic arms of the autonomic nervous system and the HF band reflects parasympathetic activity [13].

### Detrended fluctuation analysis

Detrended fluctuation analysis (DFA) quantified the correlation properties of fractal-like dynamics caused by complex interplay between vagal and sympathetic heart rate regulation [14]. The DFA technique was used to quantify the fractal scaling properties of short- and intermediate-term R-R interval time series. Clinically, reduced DFA might be more precise in predicting fatal arrhythmic events than that based on traditional methods [15]. In this study, both the short-term (DFAα1, 4 to 11 beats) and long-term (DFAα2, >11 beats) scaling exponents were calculated. All the analyses were performed by using software developed in-house provided by Matlab 7.8 (Mathworks, Inc., Natrick, Ma, USA).

### Deceleration and acceleration capacity

We used phase-rectified signal averaging method (PRSA) to process sequences of R-R interval obtained from our 30-min recordings [16]. The PRSA software was benchmarked against the freely available download from the Technical University of Munich. Deceleration capacity (DC) quantified spontaneous increases in NN intervals and acceleration capacity (AC) quantified spontaneous decrease in NN intervals. PRSA method could extract periodicities from complex time series, including noise, artefact, non-stationarities as well as periodic component. Non-periodic components were eliminated thereafter. Consequently, the PRSA computation is relatively robust against artefact and ectopic beats with extensive editing of ECG recording [17]. DC quantifies the increases in NN intervals by the phase-rectified signal averaging method, reflecting the capability to slow heart rate and AC characterized the ability to speed heart rate [16].

### Statistical analyses

The data were expressed as mean ± standard deviation. A two-way, repeated-measures analysis of variance (ANOVA) was used to evaluate statistical differences between groups (control versus CIA group) and among follow-up durations (from 1^st^ to 6^th^ week). A *p* < 0.05 was considered statistically significant and the alpha level was adjusted during multiple comparisons so as to maintain the rate of Type I error at 5% during Holm-Bonferroni post hoc analysis. When differences were found in main effect, an independent-samples *t* test was used to analyze differences between two groups at each measurement time. All analyses were performed with SPSS 19.0 (SPSS Inc. Chicago, IL).

## Results

**Fig. 1:**
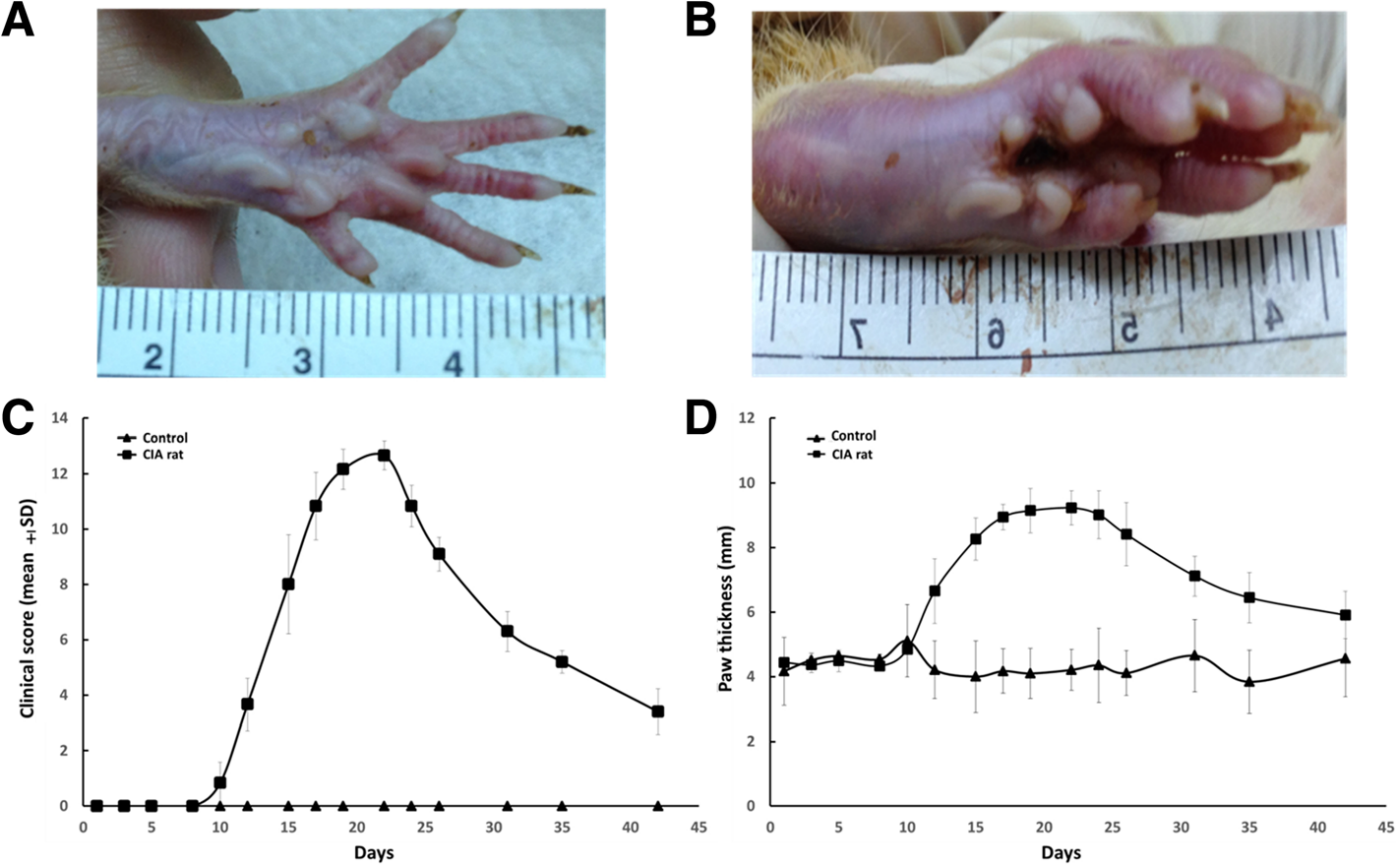
Macroscopic observation of joint swelling in collagen-induced arthritis (CIA) rats. **a** Normal rats. **b** Induced arthritis on day 20 of CIA rats. **c** The severity of arthritis in clinical scoring. **d** The severity of arthritis in paw thickness. Differences between the control group and the model group were statistically significant (*p* < 0.05)

The rats in control group were healthy and without inflammation in joints (Fig. 1a) while the rats treated with collagen were unhealthy and had progressive swelling and redness appearance in the paws (Fig. 1b). The swelling of joints in the CIA rats, especially hind paws, was gradually aggravated and approached the peak in the day 18 (Fig. 1c and d). After that, the swelling was alleviated gradually but the joints became stiff and the movements of the limbs were limited.

**Fig. 2:**
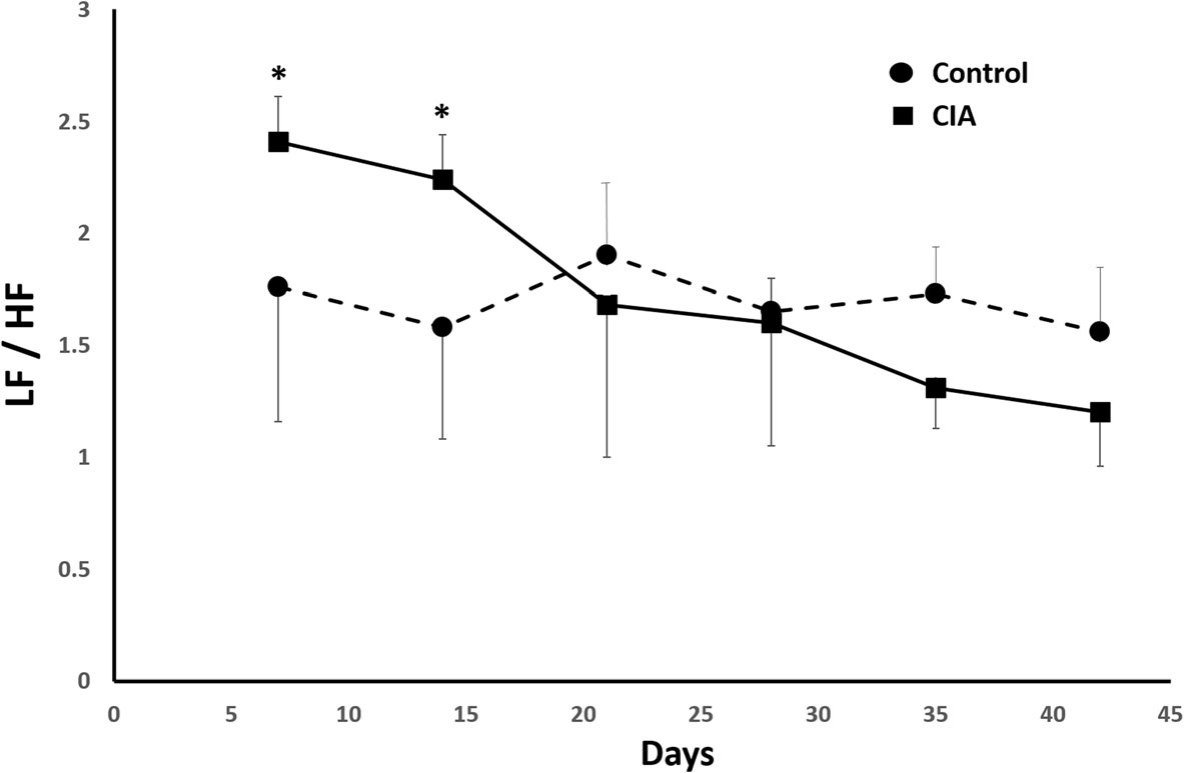
Heart rate variability of LF/HF of CIA rats and control group. Asterisk indicated *p* < 0.05 when compared with control group

**Fig. 3:**
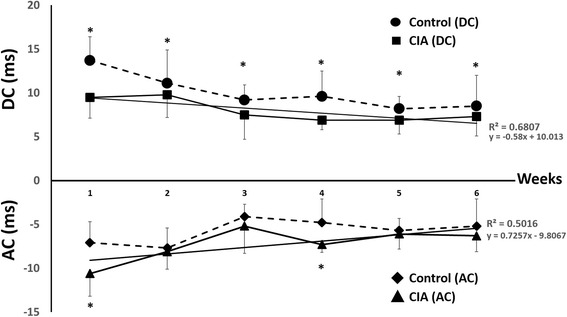
Deceleration and acceleration capacity of CIA rats and control group. The linear regression wasperformed by the least squares method. From the linear regression, the R^2^ value, the slopes and the y-intercepts are derived. Asterisk indicated *p* < 0.05 when compared with control group

The HRV parameters are shown in Table 1. The two-way repeated measure ANOVA was conducted to find out the difference HRV between groups and measurement times. It showed a significant difference among follow-up duration (*p* < 0.001), and between two groups (*p* < 0.001). Significant interaction between the groups and measurement times was shown (*p* < 0.05). In terms of liner HRV, heart rate (458 ± 35 vs. 386 ± 25, *p* = 0.002) and SDNN (8.23 ± 1.6 vs. 4.53 ± 2.5 ms, *p* = 0.004) of CIA rats were significant higher than control group in the first week. These changes persisted till 4^th^ week (2^nd^ week, heart rate: 438 ± 31 vs. 411 ± 57, *p* = 0.02; SDNN: 12.28 ± 4.3 vs. 6.08 ± 3.4 ms, *p* = 0.002 and 3^rd^ week, heart rate: 471 ± 32 vs. 411 ± 6, *p* = 0.003; SDNN: 5.82 ± 2.7 vs. 3.9 ± 0.9 ms, *p* = 0.03) when the inflammation of joins in CIA rats was relieved clinically. In frequency-domain parameters, LF (1^st^ week: 69.48 ± 5.7 vs. 61.71 ± 9.3, *p* = 0.03; 2^nd^ week: 68.41 ± 5.1 vs. 60.00 ± 7.2, *p* = 0.02 and 3^rd^ week: 68.78 ± 10.1 vs. 62.73 ± 11.1, *p* = 0.002) was significant higher in CIA rats during the first three weeks. On the other hand, HF (1^st^ week: 30.34 ± 5.6 vs. 38.01 ± 9.1, *p* = 0.02 and 2^nd^ week: 31.46 ± 5.0 vs. 39.85 ± 7.2, *p* = 0.03) was significant higher in control group during the first two weeks. In the same way, LF/HF (1^st^ week: 2.41 ± 0.7 vs. 1.76 ± 0.6, *p* = 0.01 and 2^nd^ week: 2.24 ± 0.5 vs. 1.58 ± 0.5, *p* = 0.02) of CIA rats was significant higher than control group. During 4^th^ to 6^th^week, when arthritis was relieved gradually in CIA rats, the frequency-domain parameters had no significant difference compared with control group (Fig. 2). In the DFA parameters, DFAα1 (1^st^ week: 0.88 ± 0.2 vs. 0.62 ± 0.2, *p* = 0.001; 2^nd^ week: 0.82 ± 0.2 vs. 0.74 ± 0.3, *p* = 0.02) and DFAα2 (1^st^ week: 1.12 ± 0.1 vs. 0.71 ± 0.4, *p* = 0.002; 2^nd^ week: 1.24 ± 0.1 vs. 1.09 ± 0.3, *p* = 0.03) of CIA rats were significant higher in the first two weeks. Similarly, there was no further change in the following weeks. With respect to non-linear parameters, we observed lower DC (1^st^ week: 9.5 ± 2.4 vs. 13.7 ± 2.7, *p* = 0.03; 2^nd^ week: 9.8 ± 2.6 vs. 11.1 ± 3.8, *p* = 0.03; 3^rd^ week : 7.5 ± 2.8 vs. 7.6 ± 0.7, *p* = 0.51; 4^th^ week: 6.9 ± 1.1 vs. 9.6 ± 2.9, *p* = 0.02; 5^th^ week: 8.2 ± 1.4 vs. 6.9 ± 1.6, *p* = 0.02 and 6^th^ week: 7.3 ± 2.2 vs. 8.5 ± 3.5, *p* = 0.03) in CIA rats compared with control group and there was progressive decline of DC values during whole follow up period (Fig. 3, upper panel). Among AC parameters, there was opposite trend with higher values in CIA rats (1^st^ week: -7.1 ± 2.6 vs. -10.6 ± 2.4, *p* = 0.02; 2^nd^ week: -7.7 ± 2.0 vs. -8.1 ± 6.3, *p* = 0.13; 3^rd^week : -4.1 ± 3.1 vs. -5.2 ± 0.4, *p* = 0.42; 4^th^ week: -4.8 ± 0.9 vs. -7.3 ± 2.7, *p* = 0.03; 5^th^ week: -5.7 ± 1.7 vs. -6.1 ± 1.4, *p* = 0.04 and 6^th^ week: -5.2 ± 1.8 vs. 6.3 ± 3.1, *p* = 0.33) than control (Fig. 3, lower panel)

**Table 1:** Comparison of HRV parameter between CIA rats and controls 1^st^ week to 6^th^ week

		1^st^ week	2^nd^ week	3^rd^ week	4^th^ week	5^th^ week	6^th^ week
Linear HRV parameters							
Mean HR (1/min)	Control	386 ± 25	411 ± 57	411 ± 6	377 ± 21	375 ± 18	378 ± 25
	CIA	458 ± 35*	438 ± 31*	471 ± 31*	493 ± 4^*^	383 ± 21	380 ± 28
SDNN(ms)	Control	4.53 ± 2.5	6.08 ± 3.4	3.9 ± 0.9	3.55 ± 1.3	3.89 ± 1.4	3.21 ± 1.3
	CIA	8.23 ± 1.6*	12.28 ± 4.3*	5.82 ± 2.7*	4.31 ± 1.0	5.26 ± 3.4	3.21 ± 1.3
RMSSD(ms)	Control	3.03 ± 0.8	3.35 ± 1.4	2.37 ± 0.2	2.72 ± 0.5	2.81 ± 0.5	2.62 ± 0.5
	CIA	3.37 ± 0.7	3.13 ± 0.5	2.73 ± 0.6	2.4 ± 0.1	2.44 ± 0.2	2.41 ± 0.1
LF(n.u)	Control	61.71 ± 9.3	60.02 ± 7.2	62.73 ± 11.1	60.29 ± 8.5	61.59 ± 7.9	59.01 ± 9.3
	CIA	69.48 ± 5.7*	68.41 ± 5.1*	68.78 ± 10.1*	57.89 ± 12.7	52.13 ± 16.1	49.73 ± 16.3
HF(n.u)	Control	38.01 ± 9.1	39.85 ± 7.2	37.13 ± 11	39.38 ± 8.4	38.11 ± 7.9	40.66 ± 9.1
	CIA	30.34 ± 5.6*	31.46 ± 5.0*	40.93 ± 10.1	41.77 ± 12.6	47.46 ± 15.9	49.94 ± 16.2
LF/HF	Control	1.76 ± 0.6	1.58 ± 0.5	1.9 ± 0.9	1.65 ± 0.6	1.73 ± 0.6	1.56 ± 0.6
	CIA	2.41 ± 0.7*	2.24 ± 0.5*	1.68 ± 0.4	1.60 ± 0.8	1.31 ± 0.7	1.2 ± 0.8
Non-linear HRV parameters							
DFA:α1	Control	0.62 ± 0.2	0.74 ± 0.3	0.69 ± 0.1	0.47 ± 0.1	0.49 ± 0.1	0.51 ± 0.1
	CIA	0.88 ± 0.2*	0.82 ± 0.2*	0.7 ± 0.3	0.43 ± 0.1	0.53 ± 0.1	0.52 ± 0.1
DFA:α2	Control	0.71 ± 0.4	1.09 ± 0.3	0.84 ± 0.1	0.79 ± 0.3	0.65 ± 0.2	0.52 ± 0.3
	CIA	1.12 ± 0.1^+^	1.24 ± 0.1*	0.81 ± 0.1	0.78 ± 0.1	0.62 ± 0.3	0.51 ± 0.3
DC(ms)	Control	13.7 ± 2.7	11.1 ± 3.8	9.2 ± 0.7	9.6 ± 2.9	8.2 ± 1.4	8.5 ± 3.5
	CIA	9.5 ± 2.4*	9.8 ± 2.6*	7.5 ± 2.8*	6.9 ± 1.1*	6.9 ± 1.6*	7.3 ± 2.2*
AC(ms)	Control	–10.6 ± 2.4	–8.1 ± 6.3	–5.2 ± 0.4	–7.3 ± 2.7	–6.1 ± 1.4	–6.3 ± 3.1
	CIA	–7.1 ± 2.6*	–7.7 ± 2.0	–4.1 ± 3.1	–4.8 ± 0.9*	–5.7 ± 1.7	–5.2 ± 1.8

*p < 0.05 when compared with control group using independent-samples *t* test

## Discussions

In this study, we examined various HRV parameters in a CIA rat model, which mimicking the joint inflammation of rheumatoid arthritis. We demonstrated higher time-domain parameters, LF and LF/HF ratio during acute arthritis stage (1^st^ to 3^rd^ week). There was no further difference in linear HRV parameters when the arthritis was relieved among 4^th^ to 6^th^ week. On the other hand, we observed progressive incapability of slowing and speeding heart rate in CIA rats, even after relief of joints inflammation. This is the first study to elucidate the temporal change of linear and non-linear HRV parameters in CIA rats and indicate that DC and AC could be potential surrogates of autonomic system dysregulation in patients with rheumatoid arthritis.

CIA is an induced model of rheumatoid arthritis that bears several similarities to human disease, including female vulnerability, symmetrical synovitis and inflammatory-mediated damage to the cartilage and bone of joints [18, 19]. In human RA, autonomic dysfunction were frequently observed and associated with an increased mortality risk, [7, 20] characterized by reduced HRV, an expression of sympathetic over-activity and para-sympathetic withdraw [9, 21–23]. Most of these cross-sectional studies found reduced time and frequency domain HRV parameters in RA patients when compared with control [24]. Because patients with rheumatoid arthritis had long-term disease course and several times of remission and relapsing, it was unlikely to record the temporal change of autonomic system. On the other hand, studies evaluating HRV in RA animal models are very rare. Utilizing continuous ECG recording in CIA rats and HRV analyses, our study observed the high sympathetic outflow in the acute stage of arthritis and ongoing damage of cardiac autonomic function despite of remission of arthritis.

Inflammatory cytokines and local heat and pain causes sympathetic activation by targeting the autonomic centers of the brain, consequently dampening further cytokine production and immune-inflammatory activation [25, 26]. The so-called inflammatory reflex is a self-controlling loop and an adaptive response to inflammation. However, sympathetic over-activation also affect the heart, possibly favoring onset of cardiac arrhythmia [27]. In our study, during the first three weeks after the induction, we observed increased heart rates, indicating cardiac sympathetic activation due to arthritis. There was higher SDNN in CIA rats, reflecting larger heart rate fluctuation [13, 28]. Furthermore, higher LF and LF/HF ratio in CIA rats may also represent activation of sympathetic tone. These results were opposite to most clinical trials evaluating HRV in RA patients, which revealed reduced HRV parameters. It may be because RA patients in most clinical trials had arthritis many times with chronic disease course. However, arthritis was firstly induced in our rats. In the following 4^th^ to 6^th^ week, when arthritis was relieved, the time and frequency domain parameters of CIA rats declined gradually, indicating returning to baseline sympathovagal activity.

Detrended fluctuation analysis method measures the qualitative characteristics and correlation features of heart rate behavior. Heart rate time series are fractal because they display self-similar (scale-invariant) fluctuations over a wide range of time scales [29]. These fluctuations are determined by a delicate interplay between sympathetic and vagal outflow [30]. The significant increased DFAα1 and DFAα2 in our CIA rats during first two weeks reflected increased sympathetic activity accompanied with withdraw of vagal activity [30]. Likewise, after gradual relief of inflammation in joints, both parameters had no difference compared with the control group.

As previously noted in method section, we used PRSA technique to calculate DC and AC, meaning the capability of slowing and speeding heart rates. Autonomic heart-rate modulation due to specific regulation, such as sympathovagal activity, respiratory, baroreflex mediated and circadian, occur on different timescale. PRSA technique could synchronize all periodic components of the signal irrespective of their frequency and timescale. It thus integrated all contributions of above mentioned mechanisms by accumulation the amplitude at the center of PRSA signal. Briefly, DC indicates the overall deceleration capacity of sinus rhythm, without being linked necessarily to one particular physiological process [31]. In our study, we found the progressive decline of DC despite of the recovery of arthritis. One possible explanation was that other pathophysiologic mechanisms persistently affected the SA node function in CIA rats regardless of diminished impact of inflammation reflex or sympathetic activity indicated by HRV. Noteworthy, by using short-duration ECG recording rather than long-term recording, the DC measurement would not have to account for various level of activity of CIA rats [32]. There was similar trend of progressive increase of AC but not achieving significant difference. On the other hand, our findings support that DC might be a more sensitive parameter than HRV and DFA in revealing a residual activation of the inflammatory process [31]. The residual elevated mean clinical score and paw thickness were still noted 6 weeks after the induction (Fig. 1, panels c and d, day 42), suggesting the persistence to a certain degree of inflammatory activation.

Previous studies suggested inflammatory cytokines, particularly TNF-α, could promote cardiac sympathetic activation and arrhythmic events and treatment with TNF-α antagonist could alleviate sympatho-vagal imbalance [33, 34]. HRV impairment was significantly associated with disease duration, activity and inflammation markers [35, 36]. However, few study evaluated the association of HRV impairment and cardiovascular outcomes in RA population [37], although growing evidences indicate increased cardiovascular mortality [38, 39]. In addition to conventional risk predictors of HRV proposed by the guideline [13], DC is a good predictor of morality of survivors after myocardial infarction [31]. We also demonstrated that DC is a sensitive markers of cardiac autonomic dysfunction in CIA rats.

### Limitation

There were some limitations of this study. Firstly, our rats were not monitored everyday, leaving some data gaps although our study investigated the temporal change of HRV in CIA rats. Secondly, the conclusions derived from the relatively small numbers of control and CIA rats may require careful consideration of the assumption. Thirdly, the severity of arthritis was based on clinical score measurement, lacking histopathology of joints and inflammatory cytokines from CIA rats. Similarly, we evaluate the activity of sympathetic system by using conventional HRV parameters, lacking baroreflex sensitivity or skeletal muscle sympathetic nerve activity.

## Conclusions

Our study demonstrated for the first time the dynamic change of sympathetic activity by using HRV analyses. After relief of arthritis and decline of sympathetic activity, the damage to cardiac autonomic function persisted which reflected by the reduced DC. Using these observations, more in-depth studies could be carried on to evaluate the association of autonomic dysfunction and cardiovascular events in human RA population.

## Additional file

Additional file 1: The self-made platform for ECG recording. (JPG 230 kb)

## Abbreviations

AC: Acceleration capacity
CIA: Collagen-induced arthritis
DC: Deceleration capacity
DFA: Detrended fluctuation analysis
HF: High frequency
HRV: Heart rate variability
LF: Low frequency
PRSA: Phase-rectified signal averaging method
RA: Rheumatoid arthritis
SCD: Sudden cardiac death
