# Supplementary figures and images for: Proarrhythmic risk and determinants of cardiac autonomic dysfunction in collagen-induced arthritis rats
Source: BMC Musculoskelet Disord. 2016 Nov 29;17:491. doi: 10.1186/s12891-016-1347-6 (PMC5127040; doi:10.1186/s12891-016-1347-6)

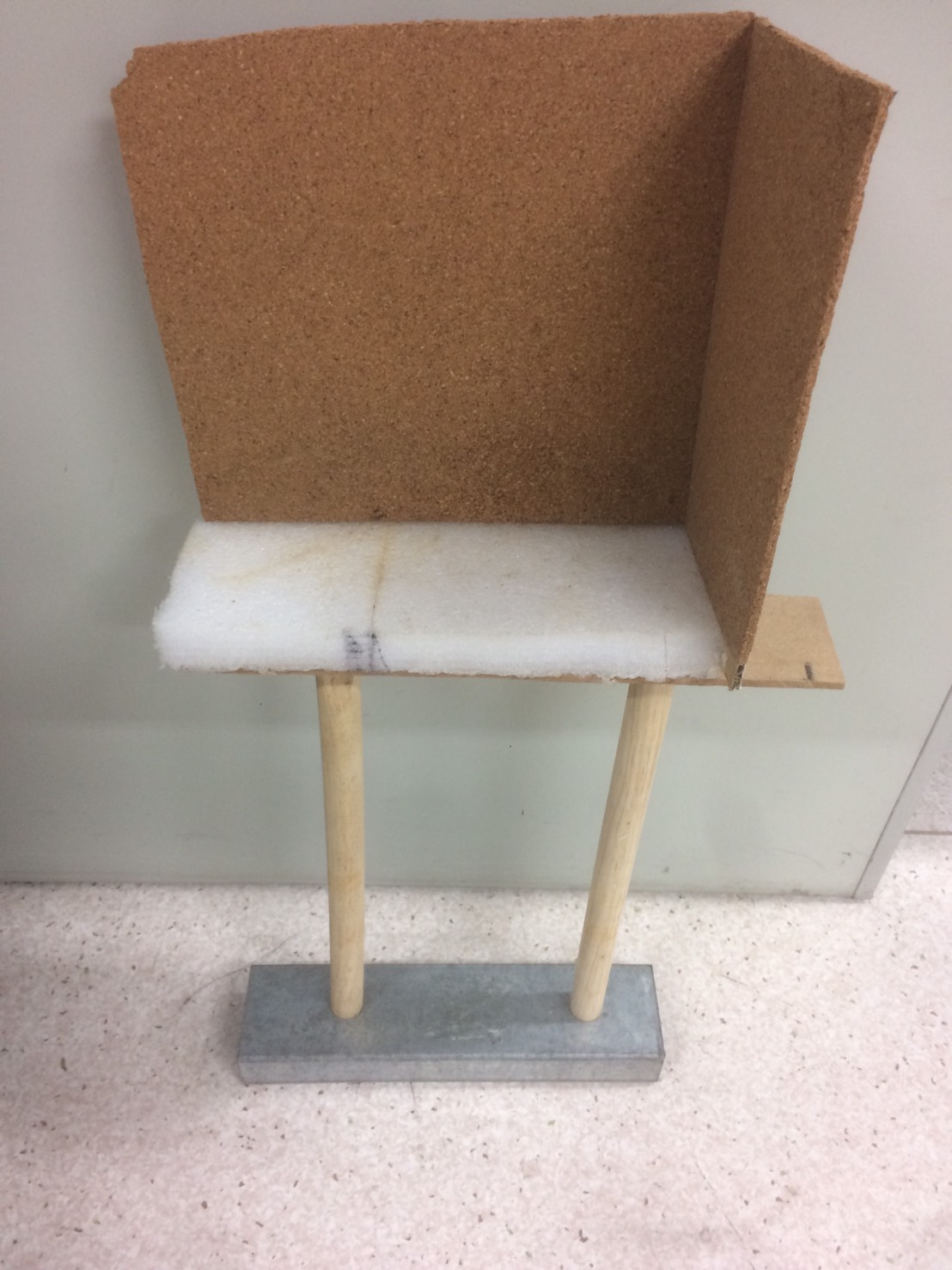

Supplement: Additional file 1: — The self-made platform for ECG recording. (JPG 230 kb) [file 12891_2016_1347_MOESM1_ESM.jpg]
